# Supplementary material for: Diindolylmethane Inhibits Cadmium-Induced Autophagic Cell Death via Regulation of Oxidative Stress in HEL299 Human Lung Fibroblasts
Source: Molecules. 2022 Aug 16;27(16):5215. doi: 10.3390/molecules27165215 (PMC9414701; doi:10.3390/molecules27165215)
Supplement: Supplementary file 1 [file molecules-27-05215-s001.zip › molecules-1798808-supplementary.pdf]

SUPPLEMENTAL MATERIAL

1 Table

2 Figures

Supplemental Table S1. List of antibodies used in the study.

Antibody	Vendor	Catalog number
P62	Santa cruz	sc-48402
Keap1	Santa cruz	sc-365626
GRP78	Santa cruz	sc-13539
Nrf2	Santa cruz	sc-13032
Lamin B1	Santa cruz	sc-374015
p-AMPKα (Thr172)	Cell signaling	2531
AMPKα	Cell signaling	2532
LC3B	Cell signaling	2775
CHOP	Cell signaling	2895
HO-1	Cell signaling	70081
Catalase	Cell signaling	12980
α-Tubulin	Cell signaling	2144
Actin	Sigma	A1978
Anti-rabbit IgG-HRP	Cell signaling	7074
Anti-mouse IgG-HRP	Cell signaling	7076

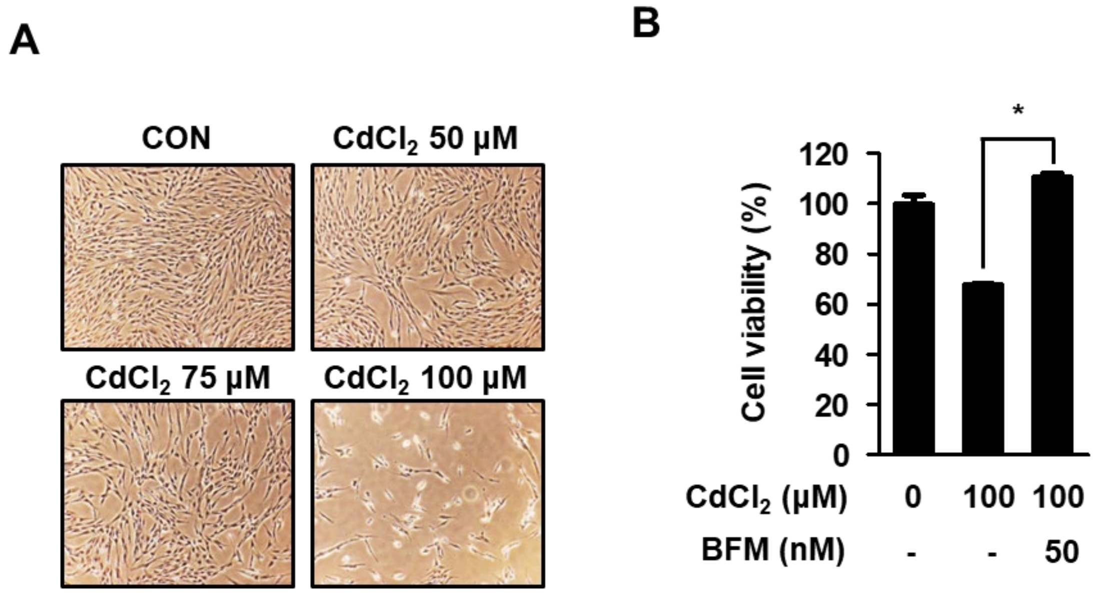

Supplemental Figure S1. Cadmium induces cytotoxicity via autophagic cell death. A, Cells were treated with the indicated concentration of Cd for 24h, and cell viability was measured using an microscopy (x100 magnification) as described in the Material and Methods. B, Cells were treated with BFM (bafilomycin A, autophagy inhibitor) in presence of Cd (100 μM), and cell viability was measured using an MTT assay. All results are presented as means ± SE of three experiments. *$P < 0.05$ vs. control.

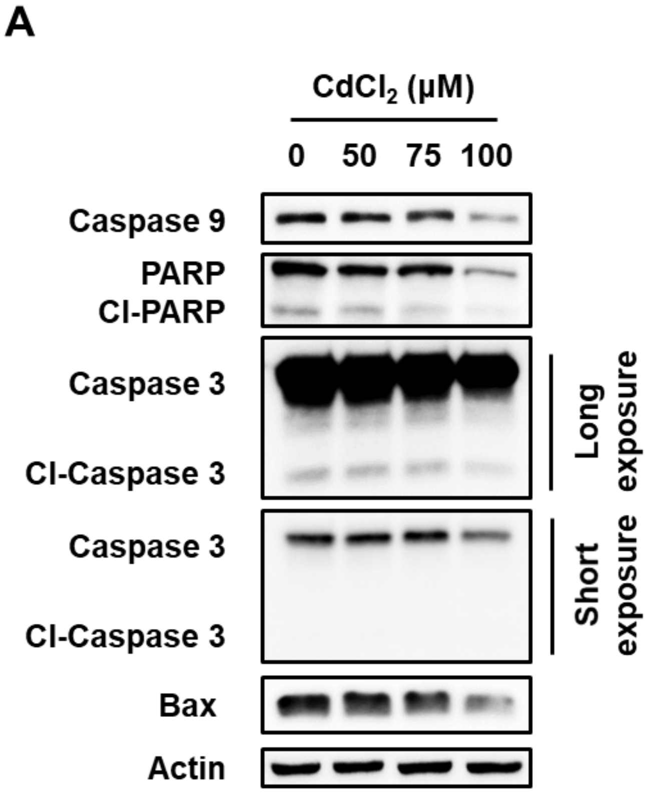

Supplemental Figure S2. Effects of cadmium on protein expression of apoptosis markers in HEL299 cells. A, Cells were treated with the indicated concentration of Cd for 24h, and whole cell lysates were analyzed by western blot analysis. Actin was used as a loading control. All results are presented as means ± SE of three experiments.
